# The development of the Dutch “National model integrated care for childhood overweight and obesity”

**DOI:** 10.1186/s12913-023-09284-z

**Published:** 2023-04-12

**Authors:** J. Halberstadt, L. W. Koetsier, M. Sijben, J. Stroo, M. van der Velde, E. G. A. H. van Mil, J. C. Seidell

**Affiliations:** 1 Department of Health Sciences, Faculty of Science, Vrije Universiteit Amsterdam, Amsterdam Public Health Research Institute, Amsterdam, The Netherlands; 2 Sijben Advies, Veghel, The Netherlands; 3 Department of Healthy Living, Public Health Service (GGD), City of Amsterdam, Amsterdam, The Netherlands; 4 Department of Paediatrics, Jeroen Bosch Hospital, ’s-Hertogenbosch, The Netherlands; 5 Maastricht University, Brightlands Campus Greenport, Venlo, The Netherlands

**Keywords:** Network care, Paediatric obesity, Primary health care, Social health care, Management childhood obesity, Sustainable behavioural change, Evidence- and practice-based model, Facilitating policy and practice

## Abstract

**Background:**

Childhood obesity is a chronic disease with negative physical and psychosocial health consequences. To manage childhood overweight and obesity, integrated care as part of an integrated approach is needed. To realise implementation of this integrated care, practical guidance for policy and practice is needed. The aim of this study is to describe the development of a Dutch national model of integrated care for childhood overweight and obesity and accompanying materials for policy and practice.

**Methods:**

The development of the national model was led by a university-based team in collaboration with eight selected Dutch municipalities who were responsible for the local realisation of the integrated care and with frequent input from other stakeholders. Learning communities were organised to exchange knowledge, experiences and tools between the participating municipalities.

**Results:**

The developed national model describes the vision, process, partners and finance of the integrated care. It sets out a structure that provides a basis for local integrated care that should facilitate support and care for children with overweight or obesity and their families. The accompanying materials are divided into materials for policymakers to support local realisation of the integrated care and materials for healthcare professionals to support them in delivering the needed support and care.

**Conclusions:**

The developed national model and accompanying materials can contribute to improvement of support and care for children with overweight or obesity and their families, and thereby help improve the health, quality of life and societal participation of these children. Further implementation of the evidence- and practice-based integrated care while evaluating on the way is needed.

## Background

### Urgency

Childhood obesity is a widespread chronic disease [1–5] with consequences for the physical and psychosocial health of both growing children (0–19 years) and adults [6, 7]. Childhood overweight and obesity also contribute to socio-economic inequalities in low- as well as high-income societies [8–10], as a lower socio-economic position is associated with the development and maintenance of overweight and obesity and vice versa [10–14]. Overweight and especially obesity are associated with considerable healthcare costs and other societal costs such as productivity losses – also in the Netherlands, where the estimated average societal cost surpasses €11,000 per person per year [15].

**Fig. 1:**
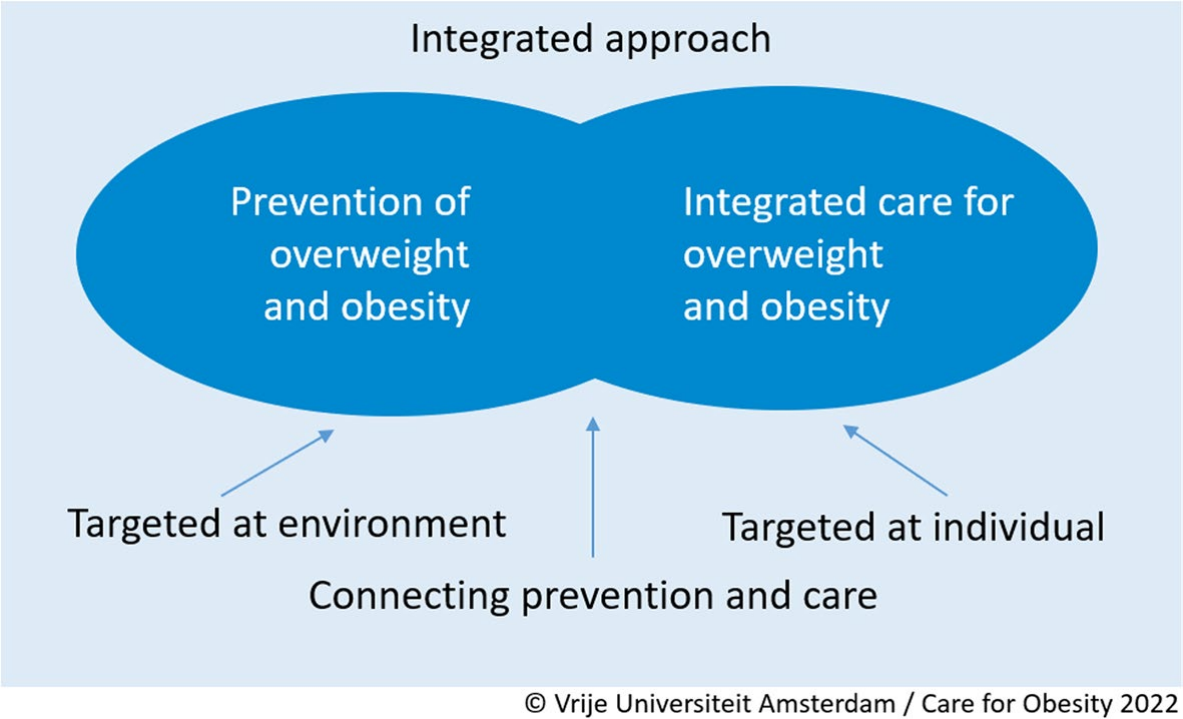
Integrated care as part of an integrated approach

For these reasons, prevention and management of overweight and obesity in children is widely recognised as important. The broadly shared view is that management of childhood obesity should be in the form of integrated care as part of an integrated approach that combines collective prevention and individual care [5, 16–19] (Fig. 1). This integrated care is also called network care or an integrated care chain, and can be defined as different organisations and professionals collaborating in a network aimed at offering optimal and coordinated support and care to a specific target group, with the right support and care at the right moment by the right professional, adapted to the requested help or care. In this case it is for children with overweight or obesity. The integrated care can be offered in the context of the social domain as well as the healthcare domain.

### History in the Netherlands

After the impactful report ‘Obesity: preventing and managing the global epidemic’ by the World Health Organization in 2000 [20] and the publication of recommendations on overweight and obesity by the Health Council of the Netherlands stating that obesity is a chronic disease [4, 21], activities around this topic intensified in the Netherlands. From 2002 the Dutch Ministry of Health, Welfare and Sport (VWS) facilitated several initiatives on prevention of and health care for overweight and obesity in adults and children.

First, the national Knowledge Centre Overweight (KCO) was established in 2002 as a platform with experts in the field of overweight and obesity, aiming to enhance knowledge about prevention and treatment [22]. In 2005 a national Covenant on Overweight (CO) was set up as a public–private partnership aiming to improve a healthy environment in the settings of school, work, home and recreation [22]. In 2010 this covenant was changed into a national initiative focused on children, to connect prevention and care locally: Youth on a Healthy Weight (JOGG) [23]. In 2008 the Partnership Overweight Netherlands (PON) started as a project with organisations of healthcare providers, health insurers and patient organisations as partners [17, 18, 24]. This partnership was set up to facilitate the development and implementation of a national healthcare standard for adults and children based on the national multidisciplinary guideline for diagnosis and treatment of adults and children that had been developed between 2005 and 2008 [25]. KCO, CO, JOGG and PON were financed by the Ministry of Health, Welfare and Sport. In 2010 the national healthcare standard was published [17, 18, 24], which together with the national guideline served as the basis for the obesity guideline for general practitioners [26] and youth healthcare workers [27]. The clinical guideline described the evidence for the effectiveness of various obesity management strategies and interventions, and the standard described how optimal strategies could be implemented in practice. In the meantime, the National Health Care Institute (ZIN) determined that a combined lifestyle intervention is the preferred treatment for obesity in adults and children and should be reimbursed by national health insurance [2]. Around the same time, Youth and Family, part of the Ministry of Health, Welfare and Sport, published a report on overweight and obesity, stating that accessible and reimbursed client-centred integrated health care should be available, especially for children [28].

With the publication of the national healthcare standard in 2010 it became clear that the next step should be its implementation. This implementation was supported financially by the Ministry. It would also help realise the not-yet fully implemented reimbursement of the needed health care. In 2011–2012 PON focused on several topics, including a supplement to the healthcare standard for children with severe obesity [29]. Next, VWS decided to focus its investments from 2013 onwards on children, and financed the establishment of the project Care for Obesity, aiming to facilitate implementation of integrated care for children (0–19 years) with overweight and obesity in the Netherlands through research and valorisation activities. In 2013–2015 Care for Obesity worked on several topics, including the development of measurement instruments for health-related quality of life [30]. In 2015 ZIN published a report on the possibilities for reimbursement of care for children with overweight and obesity, stating that more concrete examples were needed on how to organise the care; this is also because of a fundamental change in the legal system requiring municipalities to play a larger role in the organisation and financing of such care for children [31]. Clearly more activities were needed to realise implementation of the needed care for childhood overweight and obesity, and an additional request was placed with the Ministry to fund Care for Obesity from 2016 onwards.

In order to facilitate implementation of the national guideline and healthcare standard for children an extra step was needed to provide more practical guidance for policy and practice, as professionals lacked information on how to apply the guideline and standard in their daily practice. VWS financed the development of a national model based on already-existing local developments. It was decided this model should be applicable to all municipalities of the Netherlands, with tailored adaptations where needed in order to make the support and care as efficient and effective as possible. The model would make lacking support and care available to all children with overweight of obesity that need it, in order to improve their current and future physical health, health-related quality of life and societal participation. This study describes the development of a Dutch national model of integrated care for childhood overweight and obesity and accompanying materials for policy and practice. This model should facilitate support and care for children with overweight or obesity and their families.

## Methods

### Funding provided by the Ministry of Health, Welfare and Sport

In 2015 funding was requested and granted for a proposal on facilitating, describing and evaluating the content, organisation and reimbursement of care, as described in the national healthcare standard, for children (0–19 years) with overweight and obesity in local pilots in four to six municipalities for 2016–2018.

The proposal was made by Vrije Universiteit Amsterdam/Care for Obesity in close collaboration with JOGG, PON and two municipalities, also with the consent of several health insurers. The two municipalities, Amsterdam and’s-Hertogenbosch, were relatively experienced with implementing the integrated healthcare standard. They had been frontrunners since 2011 and 2009, respectively, and had already exchanged information on their local approach with other municipalities and national organisations.

Part of the proposed project plan was the development of accompanying tools for project leaders and healthcare professionals, which also included professionals from the social domain. For project leaders, part of the envisioned products to be developed by VU Amsterdam in close collaboration with the participants of a learning community was a tool to support local realisation of the national model and accompanying instruments, including an instrument to monitor the implementation.

For healthcare professionals, part of the proposed project plan was implementing, evaluating and securing a scalable webtool to measure health-related quality of life (HRQOL) as part of the diagnosis, tailoring and evaluation of treatment of childhood overweight and obesity, including educational materials on HRQOL for professionals. Funding was also requested for additional related products for research and practice. The scientific basis for the national model was thus going to be strengthened by conducting scientific research projects in close collaboration with societal partners. The results of this research were to be translated into practical tools for healthcare professionals.

In the proposal it was highlighted that to realise the integrated care that was going to be developed, reimbursement of this care by the national healthcare system – in addition to what municipalities reimbursed – would be of eminent importance. This reimbursement was not part of the requested funding.

The contribution of the different municipalities that were facilitating a local pilot of integrated care was not part of the initial proposal and funding either. After selecting a total of eight municipalities, additional funding was requested and granted for their participation in 2017–2018 in a learning community that contributed to the development of the national model and its predecessor, a basic model in the two initial municipalities (Amsterdam and ‘s Hertogenbosch).

### Development led by university team

In 2016–2018, when working on developing the national model and the accompanying tool and instruments for local realisation, the team of Care for Obesity (partly the authors of this article) also worked on several related work streams regarding HRQOL [30, 32], weight stigma and self-efficacy of healthcare professionals [33, 34], discussing weight and lifestyle with children and their parents (and caregivers) [35, 36], combined lifestyle interventions [37, 38], and integration of prevention and care [23]. The authors participating in taking on the task of the national model consisted of the national project manager of Care for Obesity (JH), a dedicated project lead (MS), a senior project worker (JS), a junior project worker (LWK) and a senior adviser (JCS). There was frequent consultation among all team members of Care for Obesity, as integration of the different work streams was an important point of attention. External personnel was hired on a regular basis to support the team with tasks like crafting the content and layout of the different tools and accompanying materials, ensuring coherence, text revisions, and building a project website.

An extra body of consultation was set up for strategic advice on embedding the results of the project at the national level, keeping a close eye on relevant developments nationally and locally, discussing possible difficulties and ensuring progress of the development. This body consisted of the national project manager, the dedicated project lead, representatives from Amsterdam and’s-Hertogenbosch, and on occasion also the senior adviser and a representative of VWS.

Frequent consultations were likewise held with JOGG, to ensure the alignment of prevention and care in their community-based approach – which involves and challenges everyone in the community to tackle childhood obesity – and to inform them about the content of the developing national model. JOGG helped ensure that the knowledge from the Care for Obesity project and its coherence with the preventative activities was shared with interested municipalities and health practitioners throughout the country via training initiatives and presentations they facilitated.

### Development of a basic model

VWS requested the use of already available materials and experiences from Amsterdam and’s-Hertogenbosch to be used as starting point for the national model that would be enriched with the experiences of other municipalities. This was a basic model developed by the two cities, both of which started experimenting with local implementation of the national healthcare standard in 2011 and 2009, respectively.

An important reason for making a basic model built on the experiences in these two municipalities was that, independently and through different kinds of local collaborations, they developed a local approach that on closer view turned out to be quite similar. This made its formation into one basic model feasible. Expanding this basic model into a national model with the input of six more municipalities (Almere, Arnhem, Maastricht, Oss, Smallingerland and Zaanstad) would provide insights into the generic components of the model and components that would require local adaptations. The basic model was ready in June 2017 and was presented at a conference of Care for Obesity and JOGG. By then the other municipalities were lined up to participate.

### Collaboration in pilot with eight municipalities

For the development of the national model, Care for Obesity collaborated with eight municipalities, combining knowledge from science, policy and practice. These municipalities operate within the Dutch healthcare setting, which by law funds support and care through different sources: funding of essential medical care is covered by the Health Insurance Act, while the funding of prevention, support and care for children is largely the responsibility of municipalities [39].

#### Selection of municipalities

The selection of the pilot municipalities started in November 2015 and aimed to invite municipalities with a variety of circumstances in addition to their phase of development with local integrated care. Eventually eight municipalities were selected to start the two-year pilot, representing a variety of population figures, regional spreading over the country’s provinces, and involvement of different health insurers. Conditions to participate as pilot municipality were willingness to use the basic model as the foundation for the local design of the support and care for children with overweight and obesity, and having a locally organised and locally financed project organisation to implement the integrated care. Municipalities were also expected to already have attention for the implementation of local collective prevention (such as promoting a healthy lifestyle, e.g. making drinking water attractive, challenging children to be physically active, providing fruits and vegetables, informing children and parents about healthy sleep) as well as an interest in the connection between prevention and care.

#### Categorisation of municipalities

These municipalities were categorised into phases that were derived from the four phases of the development model for integrated care (DMIC) [40]: 1) initiative and design phase, 2) experimental and execution phase, 3) expansion and monitoring phase, and 4) consolidation and transformation phase. At the start in 2017 the municipalities (phase 1) did not have, (phase 2) only partly had, or (phase 3) had suitably set an integrated care chain in place for children with overweight and obesity as described in the national healthcare standard. They would implement the basic model – as far as this hadn’t been done yet – and later additions, with local adaptations where needed. The phase-3 municipalities (Amsterdam and’s-Hertogenbosch) would also be dedicated to developing missing parts of the model in collaboration with the other six municipalities.

The participation of municipalities in different phases of development should lead to a national model with room for local nuances based on the experiences in the participating municipalities and the available scientific knowledge. This would be accompanied by a tool for local realisation in the rest of the country and a monitoring instrument.

#### Collaboration agreements

All participating municipalities signed a collaboration agreement with Care for Obesity. This agreement stated the goal of the collaboration – developing a national model – as well as the foreseen activities, time path and financial investment from both Care for Obesity and the municipality.

The municipalities received financial support for their participation in the pilot. This investment by Care for Obesity was fully financed by the Ministry of Health, Welfare and Sport, and was composed of a yearly allowance per municipality in 2017 and 2018, with phase-1 municipalities receiving the lowest amount (€18,720) and phase-2 and phase-3 municipalities the highest (€46,800). In year 2 the phase-1 municipalities were classified as phase-2 and reimbursed accordingly. The phase-3 municipalities requested and received extra money from the Ministry to develop additional materials for practice which would support the implementation of the national model, such as a description of a local vision and a tool for the local reimbursement of integrated care for childhood overweight and obesity, development of a tool for a broad assessment by a coordinating professional as part of the integrated care, a description of a plan and tools for the first thousand days (from conception up to the age of 2 years), a description of the specialised care plan in the hospital, and a description of the role of a coordinating professional [41–43].

The municipalities were responsible for the actual local realisation of the integrated care and committed to assigning local manpower to this end at their own cost. They also committed to sharing their knowledge and experience in the use of the basic model and the developing national model, collaborating with their local health insurer and functioning as ambassador for the integrated care. The agreement was for the duration of two years, with an evaluation after one year of the delivered input from the municipalities. All agreements were extended for one more year after this evaluation.

Care for Obesity was responsible for facilitating the development of the national model and accompanying materials through, among other things, organising a learning community.

#### Learning community

A learning community was set up by the Care for Obesity team to exchange knowledge, experiences and tools between the participating municipalities that were implementing the integrated care. The learning community was organised by the members of the project team and consisted of 18 meetings with the project leaders of the participating municipalities that took place between January 2017 and November 2018. The majority of municipalities employed two project leaders to secure organisational knowledge as well as experience with the provision of support and care for children with overweight and obesity. There were additional meetings to obtain input from policymakers, local executive professionals (such as paediatricians, general practitioners and youth doctors) and health insurers.

The meetings involved a variety of working methods in order to obtain input, such as plenary sessions, small workgroups and contributions of guest speakers about specific themes. Input was also obtained from the project leaders of the participating municipalities through assignments and a digital platform specifically developed for this purpose.

The main integrated care themes discussed at the meetings were vision, process, role of local professionals (especially the coordinating professional) and funding. Other themes discussed were measuring HRQOL as part of the diagnosis, tailoring and evaluation of treatment for childhood overweight and obesity, a toolbox to facilitate the implementation of the integrated care, and tools to monitor and evaluate it [41].

#### Commitment

Besides exchanging knowledge, experiences and tools at the meetings, project leaders had an important role in creating local and regional commitment as part of the implementation process, for instance by actively involving stakeholders (health insurers, local healthcare organisations, partners from the social domain) and sharing local experiences and developments in workshops.

Nationally, Care for Obesity also paid attention to creating commitment, e.g. by organising regional conferences in collaboration with JOGG and by giving presentations for support, healthcare and policy professionals. Care for Obesity also kept in close contact with stakeholders like VWS, ZIN, PON, health insurers, professional associations, and JOGG. Municipalities working with the integral community-based JOGG approach were supported by Care for Obesity in securing the connection between prevention and care and in developing local integrated care, based on evidence and practice.

## Results

### The national model

The developed National model integrated care for childhood overweight and obesity [44, 45] sets out a structure that provides a basis for local integrated care for these children. The four components of the national model and its developed features are elaborated upon below.

**Fig. 2:**
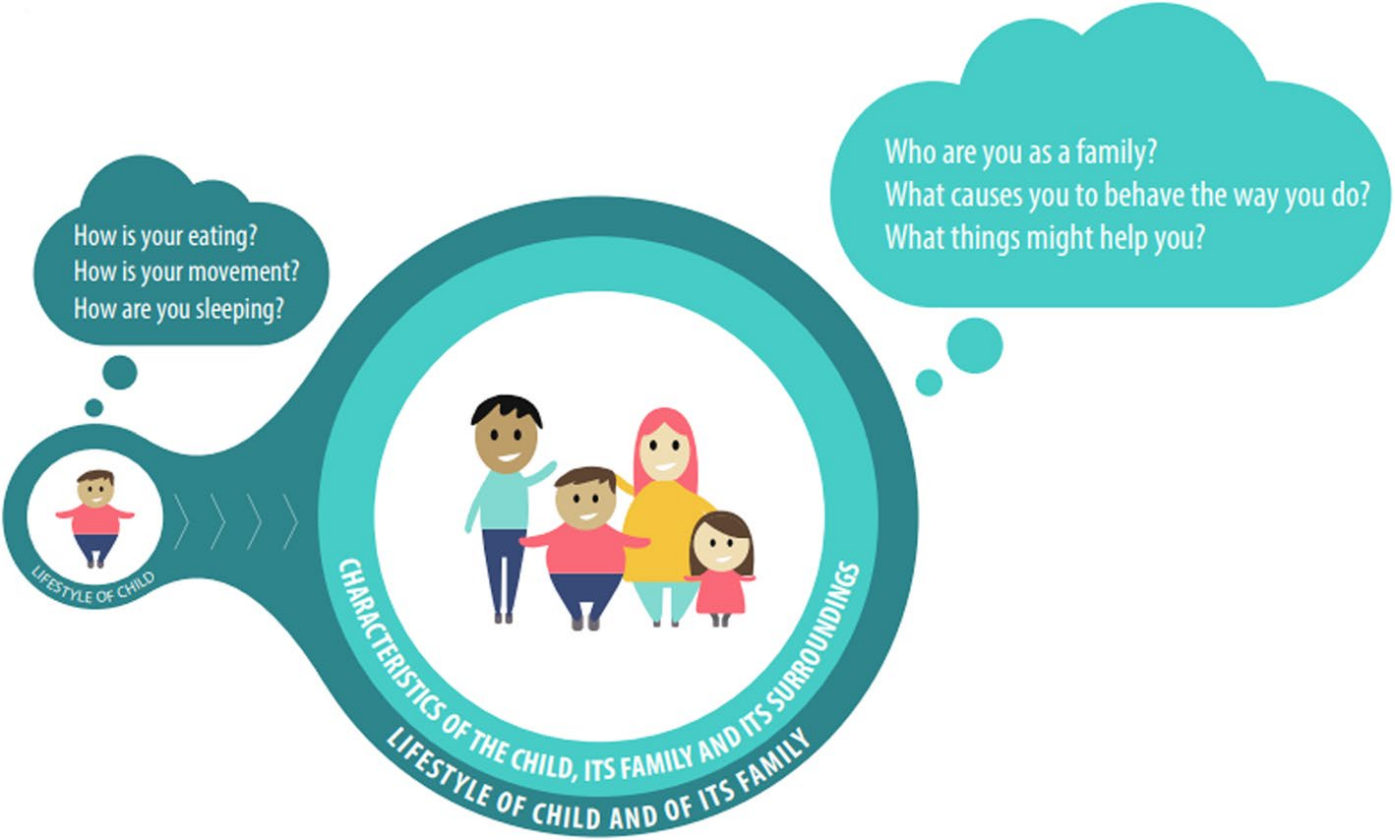
Vision of the national model: a broad perspective

**Fig. 3:**
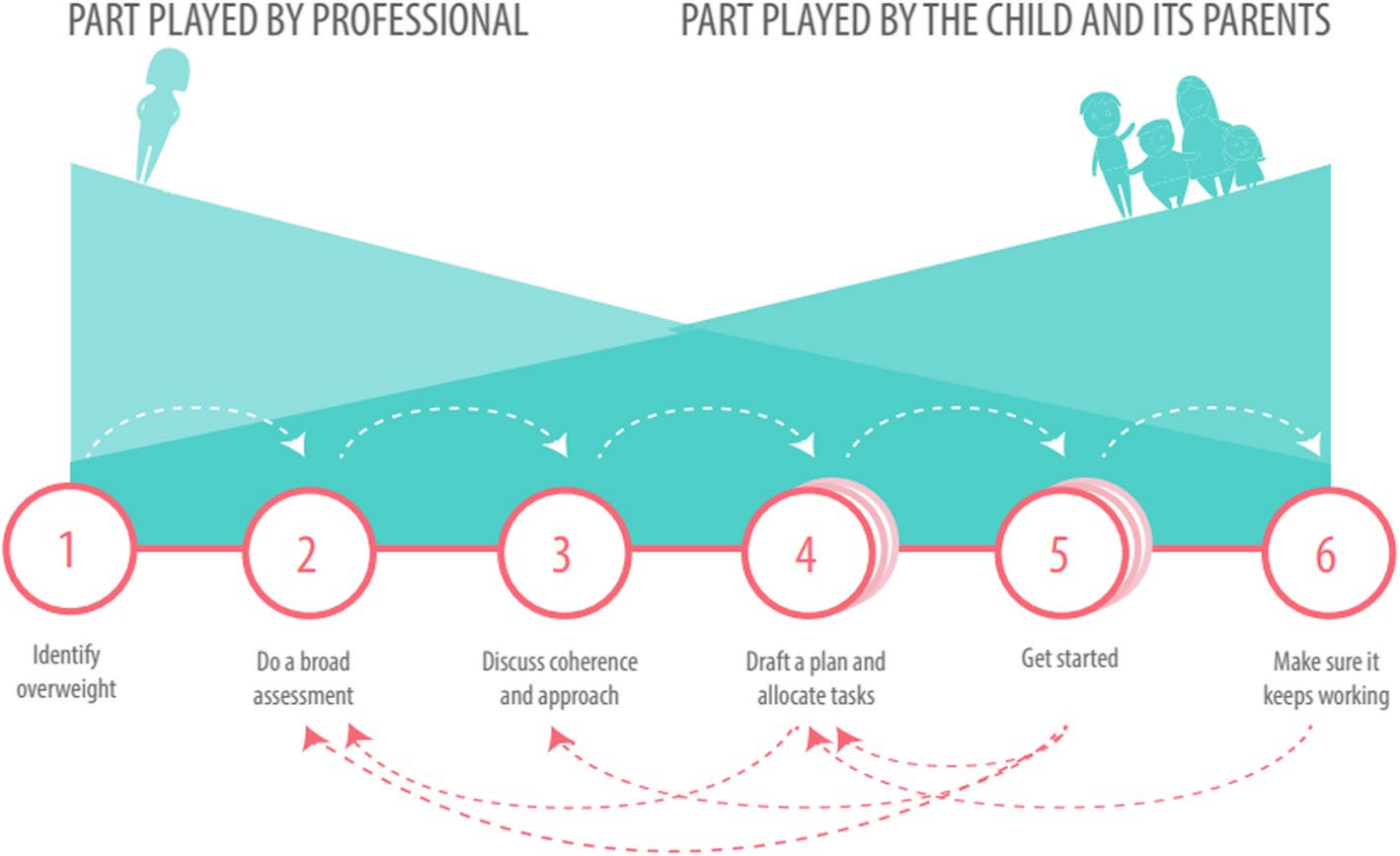
Process of the national model: a specific, clear six-step trajectory

**Fig. 4:**
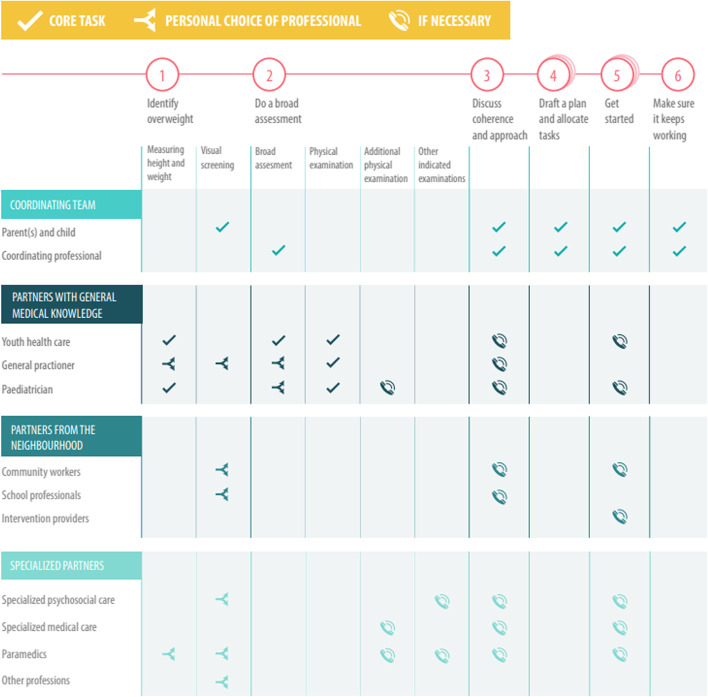
Partners of the national model: cooperation between professionals operating in the healthcare and social care domains

**Fig. 5:**
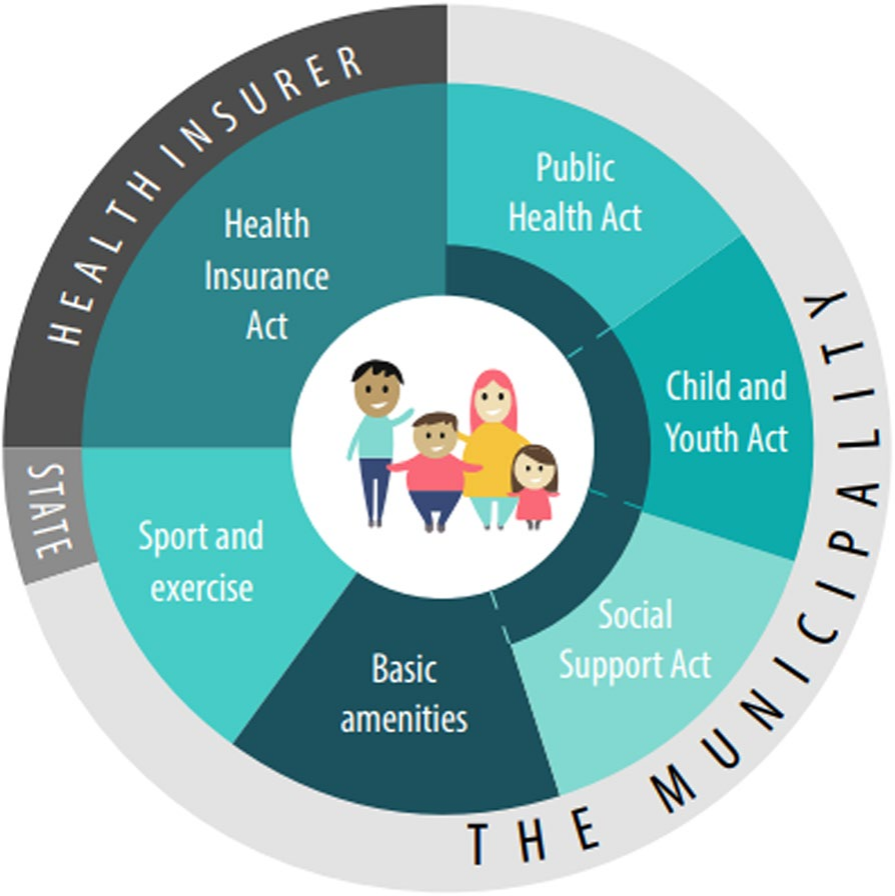
Finance of the national model: complete funding within the existing system

The vision component of the national model describes the broad perspective of the integrated care (see Fig. 2). The process component comprises a specific, clear, six-step trajectory (see Fig. 3). Although the process is similar for all children aged 0–19 years, the accompanying materials give suggestions for and room for adapting them to the age of the child. The partners component is explained by a collaboration between professionals operating in the healthcare and social care domains (see Fig. 4). The finance component is characterised by complete funding within the existing system (see Fig. 5).

### Vision

Overweight and obesity are multifactorial in origin. In other words, they are primarily triggered by behaviour that is driven by an interaction between individual biological and psychological factors and the physical, economic and socio-cultural micro and macro environment. These factors, which play a role from conception onwards, should not be seen as distinct and separate: they are closely interconnected with the well-being of the child, their parents and other significant individuals in the immediate surroundings, as well as with dynamics in the family and with the child’s social network. Hence if childhood overweight and obesity are to be dealt with effectively, these factors must be acknowledged and analysed in the context of a broad assessment. This means that biomedical factors (e.g. genetic and epigenetic factors, physical disorders or medical problems, comorbidities, extent of overweight or obesity), psychological factors (e.g. self-image, mood, well-being) and social factors of the child (at various levels, including contact with peers, school and authorities) should be assessed. Psychosocial factors of the parents (e.g. work situation, financial situation, mental and physical health, stress and traumatic events) also need to be taken into account, as well as family dynamics (e.g. family functioning and environment, parenting skills, role of co-caregivers).

Children and their parents can subsequently benefit from support in those areas of life where they are experiencing difficulties and which prevent them from improving their lifestyle. It is important that they retain as much control as possible and that they cooperate with their own social network, with the support of respectful professionals who show due care and attention to their well-being in all areas of life. Regarding the long-term improvement of behaviour – with the ultimate goal of improving the child’s health and welfare – these professionals leave matters in the hands of the family as much as possible.

The national model focuses on kick-starting the process of behavioural change based on these insights, in order to boost the chances of a long-term improvement in lifestyle. Customisation is key, and a demand-driven approach is the starting point. See Fig. 2.

### Process

The model consists of a six-step trajectory that facilitates assessing the factors causing and maintaining the overweight or obesity, and offering tailored care until child and parents have the capacity to maintain a sustainable behavioural change.

In step 1 ‘Identify overweight’, healthcare professionals take the lead in diagnosing overweight and obesity, using international cut-off points [46]. In step 2 ‘Do a broad assessment’, healthcare professionals conduct the assessment of biomedical, psychosocial and lifestyle factors in order to gain insight into the factors that may contribute to the development and maintenance of obesity and to ensure a contextualised and comprehensive understanding of children with obesity and their circumstances.

In step 3 ‘discuss coherence and approach’, the interrelatedness of factors is discussed between the coordinating professional and the child and the parents, and it is determined with which professionals to collaborate in the process and what role the child and the parents will be comfortable with. Step 4 ‘draft a plan and allocate tasks’, a support and care plan that includes agreements with the child, the parents and other professionals, is designed. Under professional supervision, the child and their family identify which health improvements (physical, psychosocial and lifestyle factors) and associated goals are desired. In step 5 ‘get started’, the support and care plan is executed, where needed with the support of professionals. In step 6 ‘make sure it keeps working’, the aim is to make sure the resulting changes are sustained. From time to time, the professional(s) involved will get in touch with the child and their family. Sometimes the behavioural changes achieved that lead to improved weight or for instance improved self-esteem of the child cannot be maintained without support. In such cases more intensive counselling is resumed, in consultation with the child and the parents.

Collaboration between professionals and the child and their family is central at every step. Healthcare professionals often play a greater role during the first three steps of the process than in the later stages. As the process advances, the family’s role increases and the involvement of the professionals decreases. This is an important precondition for ultimately achieving successful, i.e. long-term behavioural changes.

The approach is cyclical in nature. New insights can sometimes arise, or changes may occur in the surroundings or in the child which make it necessary to repeat various steps in the process. The total duration of the steps is dependent on the individual case, as the approach is tailored and depends on the request for help of the children and their family [5, 17, 18, 25, 47, 48]. See Fig. 3.

### Partners

The arenas of health and overweight and obesity are not the sole preserve of children, parents and healthcare professionals. Professionals in the social domain also have an important role to play here during all phases of the model. Support and care require effective cooperation between the child and their family and a wide range of care providers and professionals. These include social workers, educational advisers, youth care workers, community workers stimulating exercise and sports in schools and neighbourhoods, neighbourhood sports coaches, school professionals like teachers and school counsellors, youth healthcare nurses, youth healthcare doctors, general practitioners, paediatricians, paediatric nurses, dietitians, physiotherapists, psychologists and intervention providers. One of the most important elements of this model is the creation of an effective partnership between these professionals at every step of the integrated care process. This requires an additional role – the coordinating professional – whose task it is to coordinate and monitor the cohesion of all activities. This individual plays a vital role in coordinating the teamwork of the various players, motivating the child and their parents, monitoring progress, and initiating any follow-up steps that may prove necessary. These are important preconditions for success, especially where multiple problems are involved. See Fig. 4.

### Finance

The requisite support and care involves a range of different actions and actors. The funding of the different parts is based on various items of legislation [39, 44]. The funding of prevention, support and care for children is largely covered by the Youth Act and the Public Health Act (Wpg). The Health Insurance Act covers essential medical care, including the general practitioner but not mental health care for children, which is funded by the Youth Act. Other financial bases for the needed support and care are: Social Support Act (Wmo) and Dutch basic services for sports and exercise. When funding the various steps of the process, it is useful to focus primarily on the actions to be conducted and on how these are financed, rather than on the professional who performs that particular step (or part of it). In other words, content is the prime consideration and funding is ancillary. The content can thereby be based on separate components instead of a fixed total package. The individual components are brought together in order to fit with the individual situation and needs of the child and the family, making a customised approach possible. There is a financial basis for almost every part of the integrated care process, and this can differ per element. See Fig. 5.

### Accompanying materials

In addition to the national model, several materials for policy and practice were developed in 2016–2018 (with some finalised in early 2019). These can be divided into materials for policymakers to support local realisation of the integrated care as described in the national model (see Table 1. Tool to support local realisation of the national model and accompanying instruments for project leaders and Table 2. Other materials for project leaders) and materials for healthcare professionals to support them in delivering the needed support and care (see Table 3. Materials for healthcare professionals). To facilitate access to all these materials for policy and practice, they were all made available online for free and many of them were also available in print upon request.

**Table 1:** Tool to support local realisation of the national model and accompanying instruments for project leaders

Material	Description
Tool to support local realisation of the national model ^a,b^	A tool to support local realisation of the national model for project leaders that want to set up integrated care in their municipality [49]. This tool describes the phases and steps that are important in developing and implementing the approach. The tool was based on the learned lessons of the participants of the learning community, as well as the developmental model for integrated care (DMIC) [40]
Instruments accompanying the tool for local realisation *^a^	(1) A science- and practice-based module describing the reasons for monitoring the integrated care and the steps needed to set up and execute a local monitoring process [50]
	(2) An instrument to monitor local implementation of the integrated care [51], based on the developmental model for integrated care (DMIC) [40]. This instrument, a questionnaire to monitor local integrated care of childhood overweight and obesity (TICCO), has two versions: one version for professionals operating in a central position within integrated care practice (such as coordinating professionals and project leaders) and another for local executing professionals (such as youth nurses and general practitioners). The development of the TICCO is described in Koetsier et al. 2020 [52]
	(3) An overview of possible reasons for municipalities to opt for the integrated care based on what participants learned in the learning community and national advisers with experience in supporting local realisation of the approach [53]
	(4) An overview of practical obstacles when locally developing integrated care and practice-based suggestions on how to tackle these [54]
	(5) A practice-based format for a stakeholder analysis in order to map out important organisations when realising the integrated care locally [55]
	(6) A practice-based format to draw up a project plan when realising the integrated care locally [56]
	(7) A practice-based overview of relevant activities and professionals to analyse when realising the integrated care locally [57]
	(8) A practice-based profile for a project leader that can help select the right person to fulfil this role locally [58]

^*^In total eight instruments were developed, in collaboration with participants of the learning community, to supplement the tool in supporting local realisation of the national model

^a^online PDF

^b^printed document

**Table 2:** Other materials for project leaders

Material	Description
Overview of how the integrated care can be financed ^a,b^	The different elements of the integrated care were described as financed in multiple ways. Some of the care is reimbursed by health insurers, some of the support and care can be financed by legally binding responsibility of the municipalities, and some parts of the care are not financed at all or are only financed by local temporary arrangements. This overview by experts in the field provides a vision on the financing plus the different laws and possible financing sources available [39]
Description of the role of the coordinating professional ^a^	A description of characteristics of the role of the coordinating professional, including tasks to be carried out and required competences, knowledge and skills, and how the role relates to the network of other professionals and organisations [59]. This description was established on the basis of practical experiences in the municipalities of Amsterdam and ‘s-Hertogenbosch
Tool for the first thousand days ^a^	A description of how the national model can be adapted to the youngest target group (children from conception up to the age of 2 years and their parents) and specific tools to support these children in their first thousand days to prevent and reduce overweight [60–63]
Description of a local vision and a tool for local reimbursement ^a^	A description of the municipality’s vision and theoretical foundation of the local purchasing policy of integrated care for childhood overweight and obesity [64]. It aims to share the current state, practical experiences and challenges as good practice. Also a tool to determine the quality of an intervention, looking to arrive at an unambiguous vision of the quality of support and care for children with overweight and obesity [65]
Description of the specialised care plan at the hospital ^a^	A description of the position and function of the specialised care programme within the hospital but most of all within the integrated care for children with obesity. Explains which tasks, activities and responsibilities are required of the care providers [66, 67]
Animation video about the integrated care ^c^	A 5-min animated video developed by science, policy and practice experts on the highlights of the integrated care as described in the national model [68]
Summaries of the national model ^a.b^	Easy-to-read summaries in Dutch and English of the contents of the National model integrated care for childhood overweight and obesity [45, 69]

^a^online PDF

^b^printed document

^c^online animated video

**Table 3:** Materials for healthcare professionals

Material	Description
Tool for broad assessment (step 2 of the national model) ^a^	A conversation guide to conduct a broad assessment by a coordinating professional as a basis for drafting a support and care plan together with the child and family [70]. The tool provides an overview of child, family and lifestyle factors with insight into factors that might hinder or promote sustainable behavioural change. The tool is accompanied by a work instruction [71]
Webtool for measuring health-related quality of life of children with overweight and obesity ^a,b,d^	Two validated questionnaires to measure HRQOL were made available for use in the Netherlands [30]. A scalable webtool incorporating these two questionnaires was developed for measuring HRQOL as part of the tailored diagnosis and treatment of childhood overweight and obesity. The webtool was implemented, evaluated and improved [72]. A factsheet [73] based on research [32] on quality of life in treatment-seeking children with overweight, obesity and severe obesity was published
Booklet on discussing weight and lifestyle with children and parents ^a,b^	Research was conducted on several topics related to discussing weight and lifestyle: weight-based terminology in health care [35, 36], weight-biased attitudes among healthcare professionals [33], and self-efficacy and barriers of healthcare professionals [34]. Based on the research results and other available scientific literature, a booklet was developed with information on how to discuss weight and lifestyle with children and parents, including a section with information about the theoretical background of the recommendations [74]
Training initiatives on health-related quality of life, weight-biased attitudes, and discussing weight and lifestyle ^e^	Based on national and international research, and because of requests for information from practice, three training initiatives were developed and frequently given: a workshop on measuring and discussing health-related quality of life [75], a workshop on how to become aware of and handle weight-biased attitudes and stigma [76], and a workshop and multiple-day training initiative on discussing weight and lifestyle with children and their parents [77]

^a^online PDF

^b^printed document

^c^online animated video

^d^webtool

^e^live training initiatives

## Discussion

### Main findings

The aim of this study was to describe the development of the Dutch national model integrated care for childhood overweight and obesity and accompanying materials for policy and practice. The national model describes the vision, process, partners and finance of the integrated care. It thereby sets out a structure that provides a basis for local integrated care that should facilitate support and care for children with overweight or obesity and their families. The accompanying materials are divided into materials for policymakers to support local realisation of the integrated care and materials for healthcare professionals to support them in delivering the needed support and care. To our knowledge, this is the first model for implementation of reimbursed integrated chronic care for children with obesity.

### Reflections on the findings

The described Dutch model has four components (vision, process, partners, finance) which, as far as we know, have not been cohesively described like this before in the context of childhood obesity care in other countries. Still, the described care is in accordance with childhood obesity guidelines and accompanying materials for the UK, Scotland, New Zealand, the United States, Australia and Canada [78–84], as well as with the recommendations of the World Health Organization [16].

The content of the national model and accompanying materials is worth reflecting on. In contrast with the materials developed in the Netherlands, only a few countries pay attention to accompanying materials in their guidelines to support policymakers’ implementation of the guideline [78, 80, 84]. However, more is offered in other countries to support individual healthcare professionals: for example, a sensitive approach to discussing weight is widely acknowledged and other countries also have materials facilitating positive weight-related conversations [81–83].

Materials for policymakers to support local realisation of the integrated care as described in the national model and materials for healthcare professionals to support them in delivering the needed support and care seems a precondition for successful implementation of the described integrated care. The availability of these materials for a variety of professionals working at different levels (strategic, management, executive), including guidance on how they can collaborate, is a unique feature of the developed model. It should be carefully examined for each country or location where accompanying materials are needed, taking particularities into account.

An extensive description of the partners and finance as part of the national model helps realise the integrated care, since it offers a comprehensive view on what is needed for this care at the system level too. This is extra important in the Netherlands, because the support and care is financed through two domains: the social domain and the healthcare domain. The national model describes a desirable situation in which these domains strengthen each other by collaborating. Provision of support and care to families should not be hindered by miscommunications, boundaries between domains or reimbursement issues that influence the quality of the needed support and care.

The developmental process of the national model and accompanying materials yielded a number of learnings. It should be kept in mind that the development took place in the rather unique context of the Dutch healthcare system, so reproducing it in other contexts might not be easy, and the generalisability of results, learnings and recommendations outside of the Netherlands might be hampered. Still, an important implication of these findings is that the learnings regarding the process of developing such a model as well as the developed materials themselves might be applicable to other countries.

A first strength of the developmental process is that, in addition to the expertise of its project team, Care for Obesity made extensive use of science, policy and practice expertise available from other organisations. These collaborations resulted in a science- and practice-based model, thereby improving the development of the national model in all its facets. For example, it facilitated the wording of the texts in the model and all accompanying materials, making them understandable and practicable for policy and practice.

Second, the collaboration with pilot municipalities and other stakeholders also strengthened commitment to the development of a national model. This was reinforced by the local conferences that Care for Obesity and JOGG organised during the development phase.

Third, the model was developed with attention for the interests of multiple stakeholders: children and their parents/caregivers, municipalities, health insurers, healthcare professionals, JOGG, ZIN and VWS. This also contributed to the support base needed for the model and its implementation. The developmental process was likewise characterised by a carefully planned process involving multiple phases during several years and stakeholders from science, policy and practice. This automatically meant there were many interests at stake which had to be managed and many valuable views to be merged. It also meant optimal use could be made of the experience and knowledge available from all these stakeholders. By appointing a neutral national coordinator for this process – in this case a project at the university – a smooth process was facilitated and extensive support from policy and practice accomplished, resulting in an evidence- and practice-based model that was ready to be implemented beyond the eight municipalities that founded it.

Last, it seems that the procedure for the development of this model and the structure of the model can be used to develop a similar model for adults with overweight or obesity (which is in preparation), as well as models for other diseases and health/social issues in children or adults that require integrated care, such as smoking, ADHD and poverty. This implication should be further explored.

### Recommendations for research, policy and practice

Future research is needed into the effectiveness and cost-effectiveness of the integrated care described in the national model. To this end it would be useful to look into other models of integrated care for other conditions, to explore how feasible and how effective they are [85]. To gain first insights into the implementation process, a process evaluation of the local implementation was done in 2019–2021 which yielded insights into the local hindering and helping factors for implementing integrated care for children with overweight and obesity [86]. Supporting local realisation is an important task for the coming years, with the ensuing general and cost-effectiveness study. In the meantime, local monitoring can already offer insights into how the local implementation is progressing. The module developed for monitoring the integrated care is available to this end [50], and a more refined version of the indicators in this module, established with a Delphi study, was recently published [87].

It is clear that the execution of the integrated care by healthcare professionals is complicated, in part due to the complexity of the disease of obesity ([88]; van den Eynde E, Halberstadt J, Koetsier LW, Raat HJ, Seidell J, van den Akker ELT: Healthcare professionals’ perspectives on the barriers and facilitators of childhood obesity care, submitted). Additional research into the psychosocial factors causing and maintaining overweight and obesity is thus urgently needed, as well as into ways to treat children with overweight or obesity taking into account these psychosocial factors in a respectful, supportive and empowering manner. This is already being partly undertaken in the Netherlands since 2019, and has resulted in several products: a tool to assist healthcare professionals in conducting a psychosocial and lifestyle assessment [89, 90] – development of this tool is described [91, 92]; further development of the webtool to measure and discuss health-related quality of life of children with overweight and obesity [93]; supporting materials for healthcare professionals and patients [94–96] based on additional research into the needs and wishes of healthcare professionals [97]; short introductory videos to facilitate healthcare professionals’ use of these tools [98, 99]; and a folder for healthcare professionals about the words children prefer when talking to them about their weight [100], based on earlier research by Care for Obesity [36]. Additional research into potentially interacting psychosocial and lifestyle factors (at the child, family and parental level) could provide insights not only into integrated care for overweight and obesity, but also into care for a variety of other non-communicable diseases. Especially interesting to learn about seems to be the role of multi-problem cases and the implications of vulnerable situations of families on the provided support and care.

What could also help improve the integrated care for children with overweight and obesity is more research into the perspectives of children and parents, and the development of materials for them too. A start was made by Care for Obesity with a flyer containing tips for parents on how to talk to their child about a healthier weight [101]. More research, including the perspectives of children and parents on aspects like their patient journey or the role of the coordinating professionals, could provide additional insights that can be translated into advice for healthcare professionals as well as materials for families that should be developed in collaboration with healthcare professionals and families themselves.

In the meantime, further work on the implementation of the national model is required. To stimulate this in the Netherlands, the national programme Child on a Healthier Weight [102] was set up in 2019 by Care for Obesity/VU Amsterdam in collaboration with JOGG, the National Institute for Public Health and the Environment, the Netherlands Youth Institute and the Dutch Knowledge Centre Youth Health. In spring 2022 the programme was running in 40 (12%) of the total 344 municipalities in the Netherlands, including the first eight pilot municipalities. The Dutch government, which is funding this implementation, aims to reach all municipalities and thus all children that need this integrated care by 2030 [103]. To further facilitate this, the tool to support local realisation of the national model [49] was expanded and revised [104], and should be updated as new insights from practice become available. The national programme Child on a Healthier Weight took on the task to facilitate local implementation to supply them with the national model and accompanying materials through local advisers, training initiatives and a regular flow of online information targeting policymakers and healthcare professionals.

An important precondition for suitable integrated childhood obesity care is education of professionals in the vision of the national model and the ways to provide the needed care in collaboration with the other professionals in the network around a family. To this end, Child on a Healthier Weight developed a training initiative for coordinating professionals [105] that was evaluated by Care for Obesity (Koetsier LW, Boutalab L, Seidell JC, Baan CA, Halberstadt J: Training for coordinating professionals as part of the Dutch integrated care approach for childhood overweight and obesity – a mixed-method evaluation, submitted). Also, Care for Obesity developed an e-learning programme for healthcare professionals based on the previously developed training initiatives [76, 77] for discussing weight and lifestyle with children and their parents/caretakers and for handling weight-biased attitudes and stigma among healthcare professionals [106]. Adapting the training initiative for coordinating professionals to a version suitable for other groups of healthcare professionals is a logical next step, as is disseminating the available training initiatives as much as possible via channels like trade associations of healthcare professionals and regular educational programmes for healthcare professionals.

Last, an important obstacle to the implementation of integrated care for children with overweight or obesity is the lack of reimbursement of all aspects of this support and care. Hence a vital recommendation for policymakers is to sustainably organise the reimbursement of health care for children with obesity or at a high risk for this disease because of their overweight, monitoring the effects on the availability and eventually the quality of the support and care, as well as the costs. In the Netherlands the incorporation of the national model into the newly revised national guideline for the diagnosis, support and care for children and adults with obesity, whose section for children was initiated and led by Care for Obesity, should help facilitate this [5].

For all aspects of developing and implementing such a model, it is important to stay closely attuned to national developments that can help or hinder the process. In the past two decades in the Netherlands, stakeholders in science and practice as well as in policy and politics have become increasingly aware of the need for support and care for childhood obesity, and are more appreciative of it and open to optimising it. This has resulted in the Integrated Health Agreement [107] and the Healthy and Active Life Agreement [108] reached by VWS and many umbrella organisations of e.g. health insurers, municipalities, mental health services, general practitioners and patients. The aims of these agreements are accessible, qualitative, affordable support and health care based on the specific needs of people, with collaboration between the social and the healthcare domains and a healthy lifestyle among the various focal points. Part of the agreements is for health insurers and municipalities to organise integrated care for childhood overweight and obesity and four other existing integrated care initiatives for babies, adults and the elderly by 2024. The two agreements are expected to further facilitate the implementation of the national model and thereby the optimalisation of the needed support and care.

## Conclusions

Because of the high prevalence of overweight and obesity and the severe impact on and the association with the physical and psychosocial health of children in the short term and later in life, especially when it comes to obesity, support and care for these children and their families need to be improved – also because obesity is often a symptom of underlying problems that families may need support with.

The Dutch national model integrated care for childhood overweight and obesity and accompanying materials for policy and practice can contribute to this and help improve the health, quality of life and societal participation of these children. This article reflects on the learnings regarding the process of developing such a model, which might be applicable to other countries. A pilot in eight Dutch municipalities showed that implementation of the integrated care described in the developed national model is feasible and that it is supported by stakeholders from policy and practice.

The next step in the Netherlands is to further implement this evidence- and practice-based integrated care, using the described vision, partners, procedure, finance, and accompanying materials for policy and practice while evaluating on the way. This should be done in the form of integrated care as part of an integrated approach with attention for care as well as prevention.

## Data Availability

All data generated during this study are included in this published article. The data analysed during the current study are available upon reasonable request from the corresponding author (l.koetsier@vu.nl).

## References

[1] James WPT (2008). WHO recognition of the global obesity epidemic. Int J Obes.

[2] Van der Meer F, Ligtenberg G, Staal P. Preventie bij overgewicht en obesitas: de gecombineerde leefstijlinterventie [prevention for overweight and obesity: the combined lifestyle intervention]. Diemen: College voor zorgverzekeringen; 2009. Available from: https://www.zorginstituutnederland.nl/binaries/zinl/documenten/standpunten/2018/02/21/standpunt-gecombineerde-leefstijlinterventie-gli-bij-overgewicht-en-obesitas/Preventie+bij+overgewicht+en+obesitas+-+de+gecombineerde+leefstijlinterventie.pdf.

[3] American Medical Association. Recognition of obesity as a disease. American Medical Association House of Delegates Resolution; 420 (A‐13); 2013.

[4] Gezondheidsraad (2003). Dossier overgewicht en obesitas.

[5] Care for Obesity, Partnerschap Overgewicht Nederland, Nederlandse Internisten Vereniging. Guideline obesity in adults and children. Diagnostics, support and care for people with obesity or overweight combined with risk factors and/or comorbidities [Richtlijn overgewicht en obesitas bij volwassenen en kinderen. Diagnostiek, ondersteuning en zorg voor mensen met obesitas of overgewicht in combinatie met risicofactoren en/of co-morbiditeit]. Amsterdam/Rotterdam: Care for Obesity/Partnerschap Overgewicht Nederland/Nederlandse Internisten Vereniging; 2022.

[6] Sahoo K, Sahoo B, Choudhury AK, Sofi NY, Kumar R, Bhadoria AS (2015). Childhood obesity: causes and consequences. Journal of family medicine and primary care.

[7] Rankin J, Matthews L, Cobley S, Han A, Sanders R, Wiltshire HD (2016). Psychological consequences of childhood obesity: psychiatric comorbidity and prevention. Adolesc Health Med Ther.

[8] Wang Y, Lim H (2012). The global childhood obesity epidemic and the association between socio-economic status and childhood obesity. Int Rev Psychiatry.

[9] Lissner L, Wijnhoven T, Mehlig K, Sjöberg A, Kunesova M, Yngve A (2016). Socioeconomic inequalities in childhood overweight: heterogeneity across five countries in the WHO European Childhood Obesity Surveillance Initiative (COSI - 2008). Int J Obes.

[10] Bouthoorn SH, Wijtzes AI, Jaddoe VW, Hofman A, Raat H, van Lenthe FJ (2014). Development of socioeconomic inequalities in obesity among Dutch pre-school and school-aged children. Obesity.

[11] Shrewsbury V, Wardle J (2008). Socioeconomic status and adiposity in childhood: a systematic review of cross-sectional studies 1990–2005. Obesity.

[12] Anekwe CV, Jarrell AR, Townsend MJ, Gaudier GI, Hiserodt JM, Stanford FC (2020). Curr Obes Rep.

[13] Kim TJ, von dem Knesebeck O (2018). Income and obesity: what is the direction of the relationship? A systematic review and meta-analysis. BMJ Open.

[14] Gortmaker SL, Must A, Perrin JM, Sobol AM, Dietz WH (1993). Social and economic consequences of overweight in adolescence and young adulthood. N Engl J Med.

[15] Hecker J, Freijer K, Hiligsmann M, Evers S (2022). Burden of disease study of overweight and obesity; the societal impact in terms of cost-of-illness and health-related quality of life. BMC Public Health.

[16] Alleyne G, Chan Hon Yee C, Clark H, Gluckman P, Gore A, King B (2016). Report of the commission on ending childhood obesity.

[17] Seidell J, Halberstadt J, Noordam H, Niemer S. An integrated health care standard for the management and prevention of obesity in The Netherlands. Fam Pract. 2012;29(suppl 1):i153–6. 10.1093/fampra/cmr057 22399546

[18] Seidell JC, Halberstadt J, Niemer S, Noordam H (2010). Zorgstandaard Obesitas.

[19] Brownell KD (2010). The humbling experience of treating obesity: Should we persist or desist?. Behav Res Ther.

[20] World Health Organization (WHO) (2000). Obesity: preventing and managing the global epidemic.

[21] Meinders A, Fogteloo J (2003). Overgewicht en obesitas; een advies van de Gezondheidsraad [Overweight and obesity; recommendations from the National Health Council]. Ned Tijdschr Geneeskd.

[22] Renders CM, Halberstadt J, Frenkel CS, Rosenmöller P, Seidell JC, Hirasing RA (2010). Tackling the problem of overweight and obesity: the Dutch approach. Obes Facts.

[23] Seidell JC, Halberstadt J (2020). National and local strategies in the Netherlands for obesity prevention and management in children and adolescents. Obes Facts.

[24] Halberstadt J, Seidell J, Hirasing R, Renders C, van Bolhuis A (2008). Partnerschap Overgewicht Nederland: ontwikkeling van een zorgstandaard voor overgewicht en obesitas. TSG.

[25] Seidell J, Beer A, Kuijpers T (2008). Richtlijn ‘Diagnostiek en behandeling van obesitas bij volwassenen en kinderen’. Ned Tijdschr Geneeskd.

[26] Van Binsbergen J, Langens F, Dapper A, Van Halteren M, Glijsteen R, Cleyndert G (2010). NHG-standaard obesitas. Huisarts Wet.

[27] Kist-van Holthe J, Bulk-Bunschoten A, Renders C, Hirasing R, Beltman M, Timmermans-Leenders E (2012). JGZ-richtlijn overgewicht: preventie, signalering, interventie en verwijzing van kinderen van 0–19 jaar.

[28] Ministerie van Volksgezondheid Welzijn en Sport (VWS) (2009). Nota overgewicht. Uit balans: de last van overgewicht.

[29] Halberstadt J, Seidell J (2012). Addendum ernstige kinderobesitas bij de Zorgstandaard Obesitas: Ketenzorg voor kinderen met een extreem verhoogd gewichtsgerelateerd gezondheidsrisico en hun ouders.

[30] Noordam H, Halberstadt J, Seidell J (2016). Kwaliteit van leven als uitkomstmaat in de zorg voor kinderen (4–19 jaar) met obesitas. Tijdschrift voor gezondheidswetenschappen.

[31] Latta J, van der Meer F, Boluyt N (2015). Zorgaanspraken voor kinderen met overgewicht en obesitas: een handreiking.

[32] Van der Voorn B, Camfferman R, Seidell JC, Halberstadt J. Health-related quality of life in children under treatment for overweight, obesity or severe obesity: a cross-sectional study in the Netherlands. (Accepted). 10.1186/s12887-023-03973-8 PMC10088296 37038145

[33] Van der Voorn B, Camfferman R, Seidell JC, Puhl RM, Halberstadt J. Weight-biased attitudes about pediatric patients with obesity in Dutch healthcare professionals from seven different professions. J Child Health Care. 2023;0(0). 10.1177/13674935221133953 PMC10240630 36861392

[34] Van der Voorn B, Camfferman R, Seidell JC, Halberstadt J. Talking with pediatric patients with overweight or obesity and their parents: self-rated selfefficacy and perceived barriers of Dutch healthcare professionals from seven disciplines. BMC Health Serv Res. 2022:22(1236). 10.1186/s12913-022-08520-2 PMC9541008 36203179

[35] van Maarschalkerweerd PEA, Camfferman R, Seidell JC, Halberstadt J (2021). Children's, parents' and healthcare professionals' preferences for weight-based terminology in health care. Health Commun..

[36] Stuij M, van Maarschalkerweerd PE, Seidell JC, Halberstadt J, Dedding C. Youth perspectives on weight‐related words used by healthcare professionals: A qualitative study. Child: Care, Health and Development. 2020;46(3):369–80. 10.1111/cch.12760 32037594

[37] Grootens-Wiegers P, van den Eynde E, Halberstadt J, Seidell JC, Dedding C (2020). The ‘Stages towards Completion Model’: what helps and hinders children with overweight or obesity and their parents to be guided towards, adhere to and complete a group lifestyle intervention. Int J Qual Stud Health Well Being.

[38] Van den Eynde E, Camfferman R, Putten LR, Renders CM, Seidell JC, Halberstadt J (2020). Changes in the Health-Related Quality of Life and Weight Status of Children with Overweight or Obesity Aged 7 to 13 Years after Participating in a 10-Week Lifestyle Intervention. Child Obes.

[39] Sijben M, van der Velde M. Financieringsbijlage bij Landelijk model ketenaanpak voor kinderen met overgewicht en obesitas Amsterdam: Care for Obesity; 2018; Available from: https://assets.vu.nl/d8b6f1f5-816c-005b-1dc1-e363dd7ce9a5/7d450943-f1f0-4274-8991-f0555ec57fe1/Financieringsbijlage_digitaal_tcm235-928594.pdf.

[40] Minkman MM. Developing integrated care. Towards a development model for integrated care. Int J Integr Care. 2012;12(e197).

[41] Vrije Universiteit Amsterdam. Kennisexpert Kinderobesitas, Care for Obesity, Publicaties Amsterdam: Care for Obesity; Available from: https://vu.nl/nl/over-de-vu/onderzoeksinstituten/care-for-obesity/meer-over/publicaties-care-for-obesity.

[42] Gemeente Amsterdam. Professionals Sociaal Domein, Aanpak Gezond Gewicht Amsterdam: Gemeente Amsterdam; Available from: https://www.amsterdam.nl/sociaaldomein/aanpak-gezond-gewicht/.

[43] Jeroen Bosch Ziekenhuis. Proeftuin Overgewicht 's-Hertogenbosch 's-Hertogenbosch: Jeroen Bosch Ziekenhuis; Available from: https://www.jeroenboschziekenhuis.nl/proeftuin-overgewicht-s-hertogenbosch.

[44] Sijben M, Velde van der M, Mil van E, Stroo J, Halberstadt J. Landelijk model ketenaanpak voor kinderen met overgewicht en obesitas Amsterdam: Care for Obesity; 2018; Available from: https://assets.vu.nl/d8b6f1f5-816c-005b-1dc1-e363dd7ce9a5/a3efa086-7ee8-4e06-b5c9-18992dfb6bff/Landelijk_model_digitaal_tcm235-928414.pdf.

[45] Halberstadt J, Sijben M. Summary National model Integrated care for childhood overweight and obesity Amsterdam: Care for Obesity; 2019; Available from: https://assets.vu.nl/d8b6f1f5-816c-005b-1dc1-e363dd7ce9a5/8b283ede-d5fc-4bcb-978f-c693c4cf022c/Samenvatting_landelijk_model_ENG_290319_tcm235-928597.pdf. 10.1186/s12913-023-09284-z PMC10091628 37046336

[46] Cole TJ, Lobstein T (2012). Extended international (IOTF) body mass index cut-offs for thinness, overweight and obesity. Pediatr Obes.

[47] Kwaliteitsinstituut voor de Gezondheidszorg (CBO). Richtlijn Diagnostiek en behandeling van obesitas bij volwassenen en kinderen Alphen aan den Rijn: Van Zuiden Communications; 2008. Available from: https://www.nvog.nl/wpcontent/uploads/2018/02/Diagnostiek-en-Behandeling-van-Obesitas-bij-volwassenen-en-kinderen-1.0-18-03-2009.pdf.

[48] Seidell J, Halberstadt, J. Samenvatting Kinderdeel ‘Richtlijn overgewicht en obesitas bij volwassenen en kinderen. Diagnostiek, ondersteuning en zorg voor mensen met obesitas of overgewicht in combinatie met risicofactoren en/of co-morbiditeit’. TSG. 2023. (Accepted).

[49] Sijben M, Koehoorn J, Stroo J, Halberstadt J (2018). Realisatie lokale ketenaanpak voor kinderen met overgewicht en obesitas; een handreiking voor initiatiefnemers en projectleiders.

[50] Jacobs M, Sijben M, Halberstadt J. Module monitoring ketenaanpak voor kinderen met overgewicht en obesitas 2019; Available from: https://assets.vu.nl/d8b6f1f5-816c-005b-1dc1-e363dd7ce9a5/53b51b16-fd7b-45e3-a13c-27962a8d9ddb/Module_monitoring_tcm235-928599.pdf.

[51] Jacobs M, Sijben M, Stroo J, Koetsier L, Halberstadt J, Zonneveld N, et al. Hulpmiddel handreiking: Format Quickscan ketenaanpak 2019; Available from: https://assets.vu.nl/d8b6f1f5-816c-005b-1dc1-e363dd7ce9a5/3bd68aad-923e-42a6-8133-c8a2e4230726/Quickscan_betrokkenen_projectorganisatie_tcm235-929409.pdf.

[52] Koetsier L, Jacobs M, Halberstadt J, Sijben M, Zonneveld N, Minkman M. The development of a tool to monitor integrated care for childhood overweight and obesity in the Netherlands. Journal of Integrated Care. 2020.

[53] Sijben M, Koehoorn J, Stroo J. Hulpmiddel handreiking: Overzicht van belangrijkste redenen om in te zetten op een andere aanpak voor kinderen met overgewicht Amsterdam: Care for Obesity; 2019; Available from: https://assets.vu.nl/d8b6f1f5-816c-005b-1dc1-e363dd7ce9a5/0fd11a4f-6410-4a89-8c08-746ed5df4153/Overzicht_belangrijkste_redenen_om_in_te_zetten_op_een_andere_aanpak_tcm235-929408.pdf.

[54] Sijben M, Koehoorn J, Stroo J. Hulpmiddel handreiking: Overzicht mogelijke verandervraagstukken. Amsterdam: Care for Obesity; 2019; Available from: https://assets.vu.nl/d8b6f1f5-816c-005b-1dc1-e363dd7ce9a5/82edc7f9-9a39-4dd6-894f-a2bafff0cee9/Overzicht_voorbeelden_verandervraagstukken_ketenaanpak_tcm235-929407.pdf.

[55] Koehoorn J, Sijben M, Stroo J. Hulpmiddel handreiking: Format stakeholdersanalyse. Amsterdam: Care for Obesity; 2018; Available from: https://assets.vu.nl/d8b6f1f5-816c-005b-1dc1-e363dd7ce9a5/6740165f-2e15-4198-9d82-0627e8b6a786/Format_stakeholdersanalyse_tcm235-930263.pdf.

[56] Koehoorn J, Sijben M, Stroo J. Hulpmiddel handreiking: Format projectplan. Amsterdam: Care for Obesity; 2018; Available from: https://assets.vu.nl/d8b6f1f5-816c-005b-1dc1-e363dd7ce9a5/6f291cfb-cc3b-4365-a7f0-5bccbd57f231/Format_projectplan_tcm235-930261%20%281%29.pdf.

[57] Sijben M, Stroo J. Hulpmiddel handreiking: Analyse activiteiten en professionals Amsterdam: Care for Obesity; 2018; Available from: https://assets.vu.nl/d8b6f1f5-816c-005b-1dc1-e363dd7ce9a5/cf72a546-bba2-4686-b2da-4c9b20710881/Analyse_activiteiten_en_professionals_tcm235-930260.pdf.

[58] Koehoorn J, Sijben M, Stroo J. Hulpmiddel handreiking: Profielschets projectleider Amsterdam; Care for Obesity; 2018. Available from: https://assets.vu.nl/d8b6f1f5-816c-005b-1dc1-e363dd7ce9a5/774db8eb-5fa3-4b94-b78c-3b0174e629f7/18.08.23_Steam_Demo_5_Onderzoeker_tcm235-930259.pdf.

[59] Gemeente Amsterdam en Samen Gezond ’s-Hertogenbosch. Profiel Centrale Zorgverlener, voor kinderen met Overgewicht en Obesitas Amsterdam: Proeftuinen Amsterdam en ’s-Hertogenbosch; 2018; Available from: https://www.amsterdam.nl/sociaaldomein/aanpak-gezond-gewicht/ketenaanpak-proeftuin-amsterdam/#hbb57fc60-7743-4dc9-84c5-b669eaa48678.

[60] Gemeente Amsterdam en Samen Gezond ’s-Hertogenbosch. Eerste 1000 dagen aanpak. Voorkomen of verminderen van overgewicht in de eerste 1000 dagen van het leven ’s-Hertogenbosch: Proeftuinen Amsterdam en ’s-Hertogenbosch; 2018; Available from: https://www.jeroenboschziekenhuis.nl/sites/default/files/documents/2021-09/Eerste-1000-dagen-aanpak.pdf.

[61] Gemeente Amsterdam. Handreiking - Primaire preventie van overgewicht; voorkomen door signaleren van te snelle gewichtstoename bij kinderen van 0–2 jaar Amsterdam: Proeftuin Amsterdam; 2019; Available from: https://www.captise.nl/DesktopModules/Bring2mind/DMX/API/Entries/Download?EntryId=19177&Command=Core_Download&language=nl-NL&PortalId=1&TabId=123.

[62] Gemeente Amsterdam. Handreiking prenataal huisbezoek jeugdgezondheidszorg Amsterdam Amsterdam: Proeftuin Amsterdam; 2019; Available from: https://www.captise.nl/DesktopModules/Bring2mind/DMX/API/Entries/Download?EntryId=18971&Command=Core_Download&language=nl-NL&PortalId=1&TabId=123.

[63] Samen Gezond ’s-Hertogenbosch. Eerste 1000 dagen – Risicofactorenlijst overgewicht ’s-Hertogenbosch: Proeftuin ’s-Hertogenbosch; 2018; Available from: https://www.jeroenboschziekenhuis.nl/sites/default/files/documents/2021-09/Eerste-1000-dagen-risicofactorenlijst-overgewicht.pdf.

[64] Gemeente Amsterdam. Inkoop van ondersteuning en zorg voor kinderen met overgewicht en obesitas via aanbesteding flexibel preventief aanbod jeugd Inspiratiedocument voor gemeenten Amsterdam: Amsterdamse Aanpak Gezond Gewicht; 2018; Available from: https://www.amsterdam.nl/sociaaldomein/aanpak-gezond-gewicht/ketenaanpak-proeftuin-amsterdam/#ha4b1aea6-96d0-4f1a-80ba-400da5658300.

[65] Gemeente Amsterdam. Placemat kwaliteitskader: De kwaliteit van een interventie bepalen in 5 stappen Amsterdam: Amsterdamse Aanpak Gezond Gewicht; 2018; Available from: https://www.amsterdam.nl/sociaaldomein/aanpak-gezond-gewicht/ketenaanpak-proeftuin-amsterdam/#ha4b1aea6-96d0-4f1a-80ba-400da5658300.

[66] Samen Gezond ’s-Hertogenbosch. Module gespecialiseerd zorgpad voor kinderen met ernstig overgewicht ’s-Hertogenbosch: Proeftuin ’s-Hertogenbosch; 2018; Available from: https://www.jeroenboschziekenhuis.nl/sites/default/files/documents/2021-09/Expertisecentrum-overgewicht-module-gespecialiseerd-zorgpad.pdf.

[67] Gemeente Amsterdam en Samen Gezond ’s-Hertogenbosch. Adviesdocument. Bijlage bij de module Gespecialiseerd zorgpad ’s-Hertogenbosch: Proeftuinen Amsterdam en ’s-Hertogenbosch; 2019; Available from: https://www.jeroenboschziekenhuis.nl/sites/default/files/documents/2021-09/Expertisecentrum-overgewicht-adviesdocument.pdf.

[68] Sijben M, Van Mil E, Van der Velde M, Moerman I, Koetsier L, Halberstadt J. Animatiefilmpje: Ondersteuning en zorg voor kinderen met overgewicht en obesitas. Amsterdam: Care for Obesity; 2019; Available from: https://www.youtube.com/watch?v=976Unu9KPZc.

[69] Sijben M, Halberstadt J. Samenvatting Landelijk model Ketenaanpak voor kinderen met overgewicht en obesitas Amsterdam: Care for Obesity; 2019; Available from: https://assets.vu.nl/d8b6f1f5-816c-005b-1dc1-e363dd7ce9a5/ccc66a35-3085-4496-9434-0567438d1d6a/Samenvatting_landelijk_model_NL_290319_tcm235-928596.pdf.

[70] Gemeente Amsterdam en Samen Gezond ’s-Hertogenbosch. Brede anamneselijst 2–12 jaar ’s- Hertogenbosch: Proeftuinen Amsterdam en ’s-Hertogenbosch; 2018; Available from: https://www.jeroenboschziekenhuis.nl/sites/default/files/documents/2021-09/Centrale-zorgverlener-brede-anamnese-overgewicht.pdf.

[71] Gemeente Amsterdam en Samen Gezond ’s-Hertogenbosch. Werkinstructie Brede anamnese overgewicht ’s-Hertogenbosch: Proeftuinen Amsterdam en ’s-Hertogenbosch; 2018; Available from: https://www.jeroenboschziekenhuis.nl/sites/default/files/documents/2021-09/Centrale-zorgverlener-werkinstructie-brede-anamnese-overgewicht.pdf.

[72] Camfferman R, Halberstadt J. Procesevaluatie van de implementatie van kwaliteit van leven als maat in de zorg voor kinderen met overgewicht en obesitas in Nederland Amsterdam: Care for Obesity; 2019; Available from: https://assets.vu.nl/d8b6f1f5-816c-005b-1dc1-e363dd7ce9a5/43d86a4f-1cfe-4bac-9e04-56d93ac0a71e/C4O_-_Procesevaluatie_Kwaliteit_van_Leven_-_maart_2019_tcm235-928426.pdf.

[73] Camfferman R, Seidell J, Halberstadt J. Factsheet. Kwaliteit van leven van kinderen met overgewicht, obesitas en ernstige obesitas in Nederland Amsterdam: Care for Obesity; 2019; Available from: https://assets.vu.nl/d8b6f1f5-816c-005b-1dc1-e363dd7ce9a5/4e4a4673-c5c7-4674-85e6-ddb1e4176e12/C4O_-_Factsheet_kwaliteit_van_leven_van_kinderen_met_overgewicht_obesitas_en_ernstige_obesitas_-_maart_2_tcm235-928425.pdf.

[74] Niemer S, Camfferman R, van Maarschalkerweerd P, Sijben M, Seidell JC, Halberstadt J. Praten over gewicht met kinderen en ouders. Een folder voor zorg-, school-, en wijkprofessionals Amsterdam: Care for Obesity; 2019; Available from: https://assets.vu.nl/d8b6f1f5-816c-005b-1dc1-e363dd7ce9a5/cb02354c-2521-41ae-b6e8-18d184a59fa5/C4O_-_Folder_Praten_over_gewicht_-_maart_2019_tcm235-928436.pdf.

[75] Camfferman R, Noordam H (2019). Training Meten en bespreken kwaliteit van leven als onderdeel van de zorg voor kinderen met obesitas (geheel herziene versie).

[76] Camfferman R (2019). Workshop Stigma (geheel herziene versie).

[77] Gruintjes I, Niemer S, van Maarschalkerweerd PEA (2019). Training Praten over gewicht (geheel herziene versie).

[78] National Institute for Health and Care Excellence (NICE) (2014). Obesity: identification, assessment and management.

[79] Richardson L, Paulis WD, van Middelkoop M, Koes BW (2013). An overview of national clinical guidelines for the management of childhood obesity in primary care. Prev Med.

[80] Ministery of Health. Clinical Guidelines for Weight Management in New Zealand Children and Young People. Wellington: Ministery of Health; 2016.

[81] American Psychological Association. Clinical American Psychological Association. Clinical practice guideline for multicomponent behavioral treatment of obesity and overweight in children and adolescents, current state of the evidence and research needs. Washington DC: American Psychological Association; 2018.

[82] National Health and Medical Research Council (2013). Clinical practice guidelines for the management of overweight and obesity in adults, adolescents and children in Australia.

[83] Lau DC, Douketis JD, Morrison KM, Hramiak IM, Sharma AM, Ur E. Canadian clinical practice guidelines on the management and prevention of obesity in adults and children [summary]. CMAJ. 2007;176(8). 10.1503/cmaj.061409 PMC1839777 17420481

[84] Logue J, Thompson L, Romanes F, Wilson DC, Thompson J, N. S. Management of obesity: summary of SIGN guideline. BMJ. 2010;340(c154). 10.1136/bmj.c154 20181637

[85] Baxter S, Johnson M, Chambers D, Sutton A, Goyder E, Booth A (2018). The effects of integrated care: a systematic review of UK and international evidence. BMC Health Serv Res.

[86] Van Vooren N, Beijer M, Spijkerman A. Lokale implementatie aanpak Kind naar Gezonder Gewicht Bilthoven: Rijksinstituut voor Volksgezondheid en Milieu (RIVM); 2021. Available from: https://www.rivm.nl/nieuws/25-gemeenten-delen-hun-ervaringen-met-aanpak-kind-naar-gezonder-gewicht.

[87] Visscher K, Vroling H, Spijkerman A. Ontwikkeling indicatorenset Kind naar Gezonder Gewicht: een Delphi-studie. Bilthoven: Rijksinstituut voor Volksgezondheid en Milieu (RIVM). 2022. Available from: https://www.rivm.nl/documenten/ontwikkeling-indicatorenset-kind-naar-gezonder-gewicht-delphi-studie.

[88] Van Mil E, Struik A (2017). Overweight and Obesity in Children: More Than Just the Kilos. Pediatr Phys Ther.

[89] Koetsier LW, van den Eynde E, Eilander MMA, van Mil E, van der Velde M, Baan CA, et al. Leidraad voor de psychosociale en leefstijlverkenning binnen de aanpak Kind naar Gezonder Gewicht. Amsterdam: Care for Obesity; 2021; Available from: https://assets.vu.nl/d8b6f1f5-816c-005b-1dc1-e363dd7ce9a5/6a4b6442-3469-4e26-884c-88a1167e787c/JUST_VU_Care4Obesity_KnGG-stap-2-tool_brochure_DEF_20210623.pdf.

[90] Koetsier L, van den Eynde E, Eilander MMA, van Mil E, van der Velde M, Baan C, et al. Praatplaat vaststellen wat er speelt bij kind en gezin binnen de aanpak Kind naar Gezonder Gewicht Amsterdam: Care for Obesity; 2021; Available from: https://assets.vu.nl/d8b6f1f5-816c-005b-1dc1-e363dd7ce9a5/cdda1cd1-56f9-405e-834a-6631b4adca89/JUST_VU_Care4Obesity_KnGG-praatplaat-geplastificeerd_DEF_20210623.pdf.

[91] Koetsier LW, van Mil MMA, Eilander MMA, van den Eynde E, Baan CA, Seidell JC (2021). Conducting a psychosocial and lifestyle assessment as part of an integrated care approach for childhood obesity: experiences, needs and wishes of Dutch health care professionals. BMC Health Serv Res.

[92] Koetsier LW, van den Eynde E, van Mil E, van der Velde M, de Vries R, Baan CA, et al. Scoping literature review and focus groups with healthcare professionals on psychosocial and lifestyle assessments for childhood obesity care. BMC Health Services Research. 2023;23(125). 10.1186/s12913-022-08957-5 PMC9903277 36750839

[93] Eilander MMA, Halberstadt J. Webtool Meten gezondheidsgerelateerde kwaliteit van leven bij kinderen met overgewicht en obesitas. Amsterdam: Care for Obesity; 2021. Available from: https://www.kwaliteitvanlevenvragenlijsten.nl/.

[94] Eilander MMA, Koetsier LW, Halberstadt J. Handout praten over de gezondheidsgerelateerde kwaliteit van leven van kinderen met overgewicht of obesitas Amsterdam: Care for Obesity; 2021; Available from: https://www.kwaliteitvanlevenvragenlijsten.nl/wp-content/uploads/Kwaliteit-van-Leven-Vragenlijsten_Handout_bespreken.pdf.

[95] Eilander MMA, Koetsier LW, Halberstadt J. Flyer: Een vragenlijst over jouw leven. Amsterdam: Care for Obesity; 2021; Available from: https://www.kwaliteitvanlevenvragenlijsten.nl/wp-content/uploads/Kwaliteit-van-Leven-Vragenlijsten_Flyer_kind.pdf.

[96] Eilander MMA, Koetsier LW, Halberstadt J. Handleiding Meten en bespreken van de kwaliteit van leven van kinderen met overgewicht en obesitas Amsterdam: Care for Obesity; 2021; Available from: https://www.kwaliteitvanlevenvragenlijsten.nl/wp-content/uploads/Kwaliteit-van-Leven-Vragenlijsten_Handleiding_webtool.pdf.

[97] Eilander MMA, Van Mil M, Koetsier L, Seidell J, Halberstadt J. Preferences on how to measure and discuss health related quality of life within integrated care for children with obesity. Journal of Patient-Reported Outcomes. 2021;5(106). 10.1186/s41687-021-00381-3 PMC8517052 34648095

[98] Koetsier LW, Eilander MMA, Halberstadt J. Video voor gebruik leidraad en praatplaat Amsterdam: Care for Obesity; 2021; Available from: https://assets.vu.nl/d8b6f1f5-816c-005b-1dc1-e363dd7ce9a5/8cd6ee62-aaf4-4c65-b1bd-086fe25abc70/Leidraad_versie_4.mp4.

[99] Van der Voorn B, Eilander MMA, Koetsier LW, Halberstadt J. Video voor gebruik webtool meten en bespreken kwaliteit van leven Amsterdam: Care for Obesity; 2022; Available from: https://assets.vu.nl/d8b6f1f5-816c-005b-1dc1-e363dd7ce9a5/8bd9a710-3483-41a7-9700-fdbdea3a659f/Video%20Webtool%20HRQoL.mp4.

[100] Stuij M, van Maarschalkerweerd P, Dedding C, Camfferman R, Seidell J, Halberstadt J. “Deze woorden over mijn gewicht vind ik goed…” Inzichten voor zorgprofessionals die met kinderen praten Amsterdam, Utrecht: Care for Obesity, Mulier Instituut; 2021; Available from: https://assets.vu.nl/d8b6f1f5-816c-005b-1dc1-e363dd7ce9a5/ad9ce76a-f667-4532-9d80-a19d2061fa1b/Juni_2021_Folder_Deze_woorden_over_mijn_gewicht_vind_ik_goed.pdf.

[101] Eilander MMA, Koetsier LW, Halberstadt J. Hoe praat ik met mijn kind over een gezonder gewicht? Tips voor ouders en andere opvoeders Amsterdam: Care for Obesity; 2021; Available from: https://assets.vu.nl/d8b6f1f5-816c-005b-1dc1-e363dd7ce9a5/0b6f5757-8f62-4237-9c03-4bafb11663e3/Final%20praten%20met%20je%20kind.pdf.

[102] Samenwerken aan ondersteuning en zorg voor kinderen met overgewicht of obesitas: Kind naar Gezonder Gewicht (KNGG); Available from: https://kindnaargezondergewicht.nl/.

[103] Ministerie van Volksgezondheid Welzijn en Sport (VWS). Nationaal preventieakkoord [National Prevention Agreement]. Den Haag: Ministerie van VWS; 2018. Available from: https://open.overheid.nl/documenten/ronl-1f7b7558-4628-477d-8542-9508d913ab2c/pdf.

[104] Sijben M, Koehoorn J, Halberstadt J. Realisatie lokale aanpak voor kinderen met overgewicht en obesitas; een handreiking voor initiatiefnemers, projectleiders en netwerkregisseurs Amsterdam: Care for Obesity; 2021; Available from: https://assets.vu.nl/d8b6f1f5-816c-005b-1dc1-e363dd7ce9a5/ad1ed3c3-8584-4dcc-a4f5-fba8dbca190e/Handreiking%20print%202021_DEF.pdf.

[105] Hilgers A., van Riemsdijk M. Landelijk Leerlijn Centrale Zorgverleners. JOGG; 2020.

[106] Gruintjes I. E-learning Praten over gewicht Amsterdam: Care for Obesity; 2022; Available from: https://kindnaargezondergewicht.nl/praten-over-gewicht/story.html.

[107] Ministerie van Volksgezondheid Welzijn en Sport (VWS). Integraal Zorgakkoord: Samen werken aan gezonde zorg. Den Haag: Ministerie van Volksgezondheid Welzijn en Sport (VWS); 2022. Available from: https://www.rijksoverheid.nl/documenten/rapporten/2022/09/16/integraal-zorgakkoord-samen-werken-aan-gezonde-zorg.

[108] Ministerie van Volksgezondheid Welzijn en Sport (VWS). Gezond en Actief Leven Akkoord (GALA) Den Haag: Ministerie van Volksgezondheid Welzijn en Sport (VWS); 2023; Available from: https://open.overheid.nl/documenten/ronl-e8e739b2e77bf92b7bfed78d4569ae4ecbce8dac/pdf.

